# Dedifferentiated Liposarcoma of the Epididymis: A Rare Case Report and Analysis

**DOI:** 10.2174/0115734056429152260127113630

**Published:** 2026-02-17

**Authors:** Ang Li, Xukun Gao, Zhijie Wang, HaiLong Li, Yuntai Cao

**Affiliations:** 1 Department of Radiology, Affiliated Hospital of Qinghai University, Xining, China

**Keywords:** Dedifferentiated liposarcoma, Epididymis, Radiology, MRI, Diagnostic challenges, Tumor marker

## Abstract

**Introduction:**

Dedifferentiated liposarcoma is a rare type of mesenchymal sarcoma. Although most cases occur in the retroperitoneum or extremities, this report presents a rare case of dedifferentiated liposarcoma in the epididymis, highlighting the diverse sites where this disease may manifest and emphasizing the diagnostic challenges it poses, as well as the importance of comprehensive imaging and histopathological assessment.

**Case Presentation:**

*A 70-year-old Chinese male patient* presented to the urology department with progressive left scrotal enlargement, pain, and a palpable firm mass over the past month. After admission, initial ultrasound examination indicated a left spermatic cord mass. Subsequently, an enhanced MRI was performed, revealing no obvious fat signal within the lesion but significant enhancement, with thickening and compression of the spermatic cord. The findings ultimately suggested a malignant sarcoma of the left epididymis. Surgical resection and subsequent histopathological examination confirmed a spindle cell-predominant dedifferentiated liposarcoma of the epididymis encircling the spermatic cord. After 11 months of follow-up, no recurrence has been detected.

**Conclusion:**

We report a surgically confirmed rare case of dedifferentiated liposarcoma originating from the epididymis, characterized by predominant spindle cells and significant imaging enhancement. This atypical presentation complicated differentiation from other spindle cell sarcomas, highlighting diagnostic challenges at rare sites.

## 1 INTRODUCTION

Dedifferentiated liposarcoma is a rare type of mesenchymal sarcoma. The majority of cases occur in the retroperitoneum or extremities, while extremely rare cases have been reported to originate in the paratesticular region or sites such as the pancreas and gallbladder [1-3]. Most cases clinically occur in middle-aged and elderly individuals, and dedifferentiated liposarcoma exhibits highly aggressive features, including early metastasis to lymph nodes and distant organs. Currently, wide local excisionwithnegativemargins remains the only curative treatment for patients with localized dedifferentiated liposarcoma, and dedifferentiated liposarcoma exhibits poor response to currently available therapies-including radiotherapy and chemotherapy-leading to a generally poor prognosis.

## 2 CASE REPORT

### 2.1 Clinical Information

A 70-year-old Chinese male, previously in good health, presented to the urology department with gradual enlargement of the left scrotum over the past month. Physical examination revealed a mass in the left epididymis with slight tenderness on palpation. All blood tests and relevant tumor marker examinations were negative after admission.

### 2.2 Imaging Findings

**Fig. (1):**
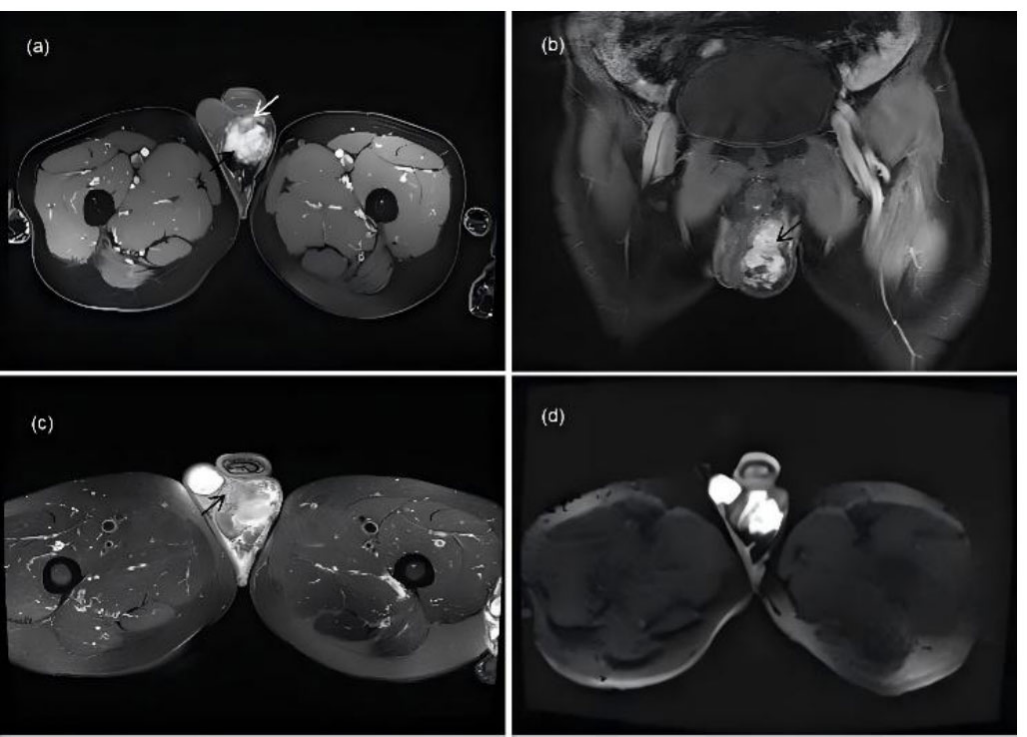
Pelvic Enhanced MRI. Axial and coronal contrast-enhanced T1-weighted images demonstrate a markedly enhancing lesion in the left epididymal region, indicated by black arrows (**a** and **b**). In the coronal view, the spermatic cord indicated by the white arrow demonstrates compression with ill-defined margins and enhancement (**a**). Axial fat-suppressed T2-weighted image shows a lesion with an isointense signal accompanied by an internal heterogeneous signal (**c**). Diffusion-weighted imaging (DWI) sequence exhibits significant restricted diffusion within the lesion (**d**).

Ultrasound examination revealed a mass in the epididymal region with thickening of the spermatic cord. Blood flow signals were observed internally and peripherally within the mass. To further clarify the nature and location of the lesion, pelvic contrast-enhanced MRI was performed: A lesion measuring 5.7 × 4.7 × 4.0 cm was identified in the left epididymis, demonstrating isointense signal on T1-weighted imaging (T1WI) and T2-weighted imaging (T2WI), with internal heterogeneous signal. The lesion showed marked enhancement on contrast-enhanced T1WI and significant diffusion restriction on diffusion-weighted imaging (DWI; b-value: 1200). The imaging revealed aggressive tumor growth with displacement of the spermatic cord and testis due to compression (Fig. **1a**-**d**).

Based on the above findings, malignant sarcoma of the left epididymis was suspected. The patient ultimately underwent a left epididymal-orchiectomy. Intraoperative findings revealed abnormal morphology of the epididymis encircling the spermatic cord, with calcified components present within the tumor tissue. During the 11-month follow-up period after the surgery, no obvious signs of recurrence have been observed so far.

### 2.3 Histopathological Findings

**Fig. (2):**
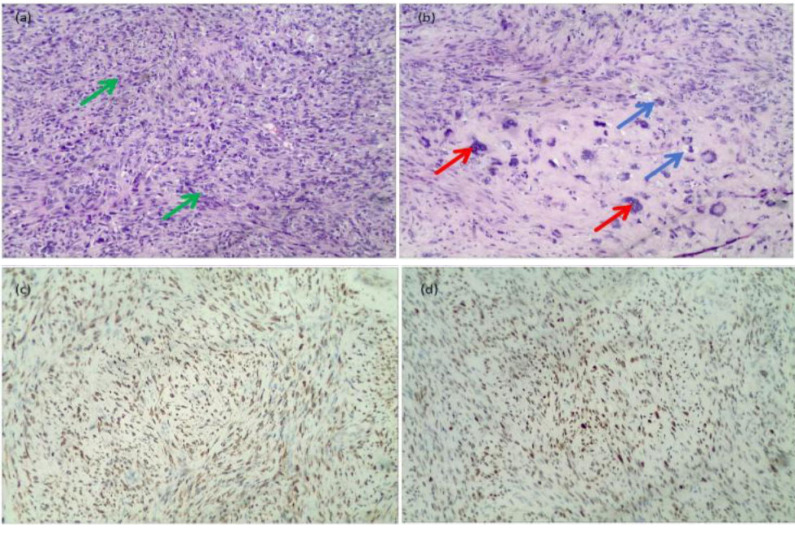
Hematoxylin and Eosin (H&E) Staining and Immunohistochemistry. Under a high-magnification H&E-stained section (×100) microscope, the tumor cells are observed to exhibit a fibrosarcoma-like morphology. The tumor cells (green arrows) are arranged in bundles, with a disordered and irregular cell arrangement. The cells show prominent atypia. Some tumor cells have distinct nucleoli. Individual lipoblasts (red arrows) and numerous tumor giant cells (blue arrows) are visible. Some nuclei are arranged in a wreath-like pattern. Focal hyaline degeneration is seen in part of the tumor stroma, with a small amount of lymphocyte infiltration in focal areas, and obvious blood vessels are present in the stroma(**a** and **b**). The results of immunohistochemical staining for CDK4 (**c**) and MDM2 (**d**) [both ×100] show that the nuclei of spindle-shaped tumor cells are stained brown, indicating positive expression, suggesting a possible lipogenic tumor. Most of the tumor cells are dedifferentiated components(**c** and **d**). Considering the diffuse positive expression of P16 and Desmin, the diagnosis is supposed to be a dedifferentiated liposarcoma.

Histopathological examination revealed tumor cells arranged in fascicular patterns, with occasional lip blasts and abundant tumor giant cells. Immunohistochemical analysis showeddiffusepositivityforCDK4,MDM2,P16,and Desmin, leading to a diagnosis ofdedifferentiatedliposarcoma(Fig. **2a**-**d**).

## 3 DISCUSSION

### 3.1 Demographics and Clinical Presentation

Liposarcoma is the most common soft tissue sarcoma, typically classified into three subtypes: well-differentiated/dedifferentiated, myxoid, and pleomorphic liposarcoma, accounting for approximately 20% of all malignant mesenchymal tumors. Well-differentiated liposarcoma and dedifferentiated liposarcoma together constitute the largest subgroup of liposarcoma. Notably, dedifferentiated liposarcoma presents as a high-grade sarcoma characterized by aggressive local growth, with significantly increased risks of local recurrence and tumor-related mortality. Clinically, dedifferentiated liposarcoma commonly occurs in middle-aged and elderly populations, with potential sites including the retroperitoneum, extremities, and scrotum. At the same time, its occurrence in superficial tissues is as low as less than 1%. Its occurrence in superficial tissues is less than 1%. The clinical presentation is typically manifested as a painless mass, usually nonspecific [4, 5].

### 3.2 Imaging Features

From an imaging perspective, myxoid liposarcoma contains minimal fat (<10%) while exhibiting extremely high water content, resulting in fluid-like low density on CT scans or high signal intensity on T2-weighted MRI. The “feathery” enhancement pattern in the myxoid lesion area serves as a significant characteristic. Pleomorphic liposarcoma shares imaging similarities with other high-grade sarcomas. On imaging, it typically presents as a large, multinodular, well-defined mass with frequent hemorrhage and necrosis, along with minimal adipose tissue. These features often result in heterogeneous signal intensity on MRI, manifesting as a complex, bulky mass.

Well-differentiated liposarcoma typically contains more than 75% adipose tissue and commonly exhibits a few hyperenhancing nodules and septated solid areas. The radiologic features of dedifferentiated liposarcoma are closely correlated with the proportion and distribution of components with varying degrees of differentiation within the tumor. It is typically a “mixed-type” tumor containing both well-differentiated and dedifferentiated components, with the key imaging feature distinguishing dedifferentiated liposarcoma from well-differentiated liposarcoma being the presence of larger and more prominent solid tumor areas, usually exceeding 1 cm in size. However, atypical dedifferentiated liposarcomas may also present with considerable complexity on imaging, characterized by heterogeneous nonlipomatous components such as abundant spindle-shaped and pleomorphic cellular elements, along with the presence of coarse, amorphous calcifications, posing diagnostic challenges in distinguishing them from entities like solitary fibrous tumor and rhabdomyosarcoma.

When morphological features suggest an aggressive soft tissue tumor and findings such as fat necrosis or calcification indicate clues suggestive of dedifferentiation, the diagnosis may point to dedifferentiated liposarcoma. Therefore, the practical application of diagnostic imaging analysis can help clinicians understand lesion composition, thereby guiding decision-making and optimizing treatment, while also aiding in avoiding unnecessary preoperative biopsies [4-7].

### 3.3 Pathological Characteristics

At the histological level, dedifferentiated liposarcoma is typically characterized by undifferentiated pleomorphic sarcoma or spindle cell sarcoma, often coexisting with well-differentiated liposarcoma. It may serve as a recurrent or de novo manifestation of well-differentiated liposarcoma. Research has revealed that the underlying genetic aberrations in dedifferentiated liposarcoma are associated with high-level amplification of the chromosomal 12q13-15 region, with oncogenes CDK4 and MDM2 being particularly prominent. Additionally, recurrent amplification of 1p32 and 6q23 is associated with poorer prognosis in dedifferentiated liposarcoma, and overexpression of ASK1 (located at 6q23) and JUN (1p32) is linked to dedifferentiation. In current pathological diagnosis work for dedifferentiated liposarcoma, the combined use of CDK4, MDM2, and p16 is relatively common and holds significant value for its differential diagnosis [5, 8, 9].

### 3.4 Treatment and Prognosis

In the realm of treatment, similar to common soft tissue sarcomas, the application of radiotherapy and chemotherapy remains a key area of research. For superficial dedifferentiated liposarcoma, relatively more studies have been conducted. Some research indicates that adding preoperative radiotherapy to patients undergoing limb-sparing surgery for superficial dedifferentiated liposarcoma results in relatively few side effects and can significantly reduce the risk of recurrence. In contrast, research on chemotherapy is less extensive; for patients with a higher likelihood of metastasis and recurrence, the use of perioperative chemotherapy can be considered as a treatment option. Compared to its superficial counterpart, deep-seated dedifferentiated liposarcoma is less common. Furthermore, due to the heterogeneity of liposarcoma in histology, molecular subtypes, and clinical features, conducting clinical trials tailored to specific sarcoma histotypes is challenging. Consequently, research on radiotherapy and chemotherapy for deep-seated dedifferentiated liposarcoma is relatively scarce [10, 11]. Regarding advanced disease management, there is still a lack of effective systemic treatment options for dedifferentiated liposarcoma. Anthracycline-based drugs remain the most commonly used first-line therapy for the systemic treatment of dedifferentiated liposarcoma [12]. Targeted agents such as MDM2 antagonists [13] and CDK4 inhibitors [14], although currently demonstrating suboptimal efficacy, are under active investigation and may potentially alter the treatment paradigm for patients with dedifferentiated liposarcoma in the future. Additionally, due to inherent resistance to both chemotherapy and radiotherapy, systemic therapy is rarely employed for localized dedifferentiated liposarcoma. Therefore, surgical resection remains the primary method for managing localized dedifferentiated liposarcoma and addressing local postoperative recurrence [5, 9].

Research on multidisciplinary management approaches for dedifferentiated liposarcoma is also rapidly emerging, highlighting the critical role of multidisciplinary collaboration in precision diagnosis and treatment for patients, while also expanding new horizons for clinical management strategies [10].

### 3.5 Study Limitations

The clinical examinations for this case are comprehensive; however, a current limitation is the relatively short follow-up period. We will continue to follow up with this patient and report the findings in a subsequent article.

### CONCLUSION

We identified and surgically confirmed a rare case of epididymal dedifferentiated liposarcoma, which posed diagnostic challenges in clinically and radiologically distinguishing it from other spindle cell sarcomas. This case study highlights that dedifferentiated liposarcoma with atypical presentations in uncommon locations should be included in the differential diagnosis of epididymal malignancies. It aims to enhance recognition of such tumors at atypical sites and underscores the critical integration of imaging and histopathological evaluation to improve diagnostic accuracy and optimize treatment planning.

## Data Availability

All the data and supporting information are provided within the article.

## LIST OF ABBREVIATIONS

T1WI: T1-weighted imaging
T2WI: T2-weighted imaging
DWI: Diffusion-weighted imaging
CDK4: Cyclin dependent kinase 4
MDM2: Mouse doubleminute 2 homolog
P16: MTS (multiple tumor suppressor 1)
ASK1: Apoptosis signal-regulating kinase 1

## STANDARDS OF REPORTING

CARE guidelines were followed.
